# Development of Magnetic Probe for Sentinel Lymph Node Detection in Laparoscopic Navigation for Gastric Cancer Patients

**DOI:** 10.1038/s41598-020-58530-5

**Published:** 2020-02-04

**Authors:** Akihiro Kuwahata, Ryo Tanaka, Sachiko Matsuda, En Amada, Tomoyuki Irino, Shuhei Mayanagi, Shinichi Chikaki, Itsuro Saito, Norio Tanabe, Hirofumi Kawakubo, Hiroya Takeuchi, Yuko Kitagawa, Moriaki Kusakabe, Masaki Sekino

**Affiliations:** 1 Graduate School of Engineering, The University of Tokyo, Tokyo, 113-8656 Japan; 2 Department of Surgery, Keio University School of Medicine, Tokyo, 160-8582 Japan; 3 Department of Surgery, Hamamatsu University School of Medicine, Hamamatsu, 431-3192 Japan; 4 iMed Japan Inc., Chiba, 275-0001 Japan; 5 Research Center for Food Safety, Graduate School of Agricultural and Life Sciences, The University of Tokyo, Tokyo, 113-8657 Japan; 6 Matrix Cell Research Institute Inc., Ibaraki, 300-1232 Japan

**Keywords:** Biomedical engineering, Electrical and electronic engineering

## Abstract

New laparoscopic sentinel lymph node navigation using a dedicated magnetic probe and magnetic nanoparticle tracer for gastric cancer patients allows minimally invasive surgeries. By identifying the sentinel lymph nodes containing magnetic nanoparticles, patients can avoid excessive lymph node extraction without nuclear facilities and radiation exposure. This paper describes the development of the laparoscopic magnetic probe, *ACDC-probe*, for laparoscopic sentinel lymph node identification utilizing the nonlinear response of the magnetic nanoparticles magnetized by an alternating magnetic field with a static magnetic field. For highly sensitive detection, the ratio of static to alternating magnetic fields was optimized to approximately 5. The longitudinal detection length was approximately 10 mm for 140 μg of iron, and the detectable amount of iron was approximately 280 ng at a distance of 1 mm. To demonstrate the feasibility of laparoscopic detection using the *ACDC-probe* and magnetic tracers, an experiment was performed on a wild swine. The gastric sentinel lymph node was clearly identified during laparoscopic navigation. These results suggest that the newly developed *ACDC-probe* is useful for laparoscopic sentinel lymph node detection and this magnetic technique appears to be a promising method for future sentinel lymph node navigation of gastric cancer patients.

## Introduction

The technique of sentinel lymph node biopsy (SLNB) was introduced to investigate cancer metastases in lymph nodes (LNs) and to avoid the unnecessary removal of LNs inducing side effects owing to the obstruction of lymphatic drainages^1–3^. Sentinel lymph nodes (SLNs) that first receive the lymphatic flow from the primary tumor site would be a likely site of metastasis and can be identified by using a dedicated detector and tracer. By only investigating metastasis inside the identified SLNs containing a dedicated tracer, surgeons obtain sufficient information for the determination of subsequent cancer treatments, and cancer patients can avoid the excessive removal of LNs, which prevent associated complications and improve the patient’s quality of life (QOL).

The current standard techniques of SLNB using a radioisotope (RI, generally technetium 99 m: ^99m^Tc) and/or infrared-fluorescence (indocyanine green: ICG) tracer, and a dedicated gamma probe and/or infrared camera probe has been established^4^. However, their methods involve some drawbacks. The RI method requires nuclear facilities and cancer patients, as well as technicians, are exposed to radiation. For the ICG method, there is a risk that a larger number of SLNs might be identified due to the high drainage of ICG compared with the RI method (a fluorescence tracer rapidly spreads to SLNs/LNs), resulting in the risk of the unnecessary removal of LNs to some extent. To overcome these drawbacks, the magnetic techniques of SLNB using magnetic tracers containing magnetic nanoparticles (MNPs) and a dedicated magnetic probe have been explored^5^ and has been sufficiently established in breast cancer patients^6–12^ as an innovative method. The additional advantage of magnetic techniques is that pre- and post- operative magnetic resonance imaging (MRI) can guide the surgeon to the correct location and can help confirm whether all SLNs were resected, respectively. Furthermore, the combination of magnetic techniques and ICG methods provide dual-tracer navigation^13^ without RI, thereby leading to more accurate SLN detection.

As with breast cancers, in the laparoscopic SLN navigation of cancer patients, there is a clinical need for the development of this magnetic technique. Laparoscopic operations are being widely performed on patients with different cancers, such as gastric cancer^14–23^, esophageal cancer, uterine cancer, colon cancer, and gallbladder cancer^24^. In particular, approximately 1 million new cases of gastric cancer were reported in 2018 worldwide, the third highest among all cancers^25^. The percentage of early gastric cancer patients (mucosal cancer or submucosal cancer) with lymph node metastases is approximately 10–15% in general. However, as surgeons cannot judge lymph node metastases before surgical dissections without laparoscopic SLN navigation, they remove the stomach and lymph nodes of all gastric patients. Thus, laparoscopic SLN detection is very important to avoid overtreatment for 85–90% of gastric cancer patients.

The structure of existing magnetic probes developed for breast cancer patients, such as *DC-probe* (developed by us)^26–29^, Sentimag® magnetometer^5,30–32^, DiffMag magnetometer^33,34^, fluxgate magnetometer^35^, and a magnetometer based on magnetic tunneling junction sensors^36^, is not applicable for SLN detection in laparoscopic surgery because the typical surgical instruments of laparoscopic surgery are inserted inside the patient’s body through a port (a trocar) with an inner diameter of 12 mm; currently, the outer diameter of all probes is larger than 12 mm. Furthermore, eliminating the combined influences from other medical instruments, such as forceps and a laparoscopic camera, is required to more precisely detect SLNs in the limited intra-abdominal space. Considering the principles of magnetic detection using only AC or DC (*DC-probe*) magnetic fields, their detection can be easily affected by the other medical instruments, which means the precise detection of tiny MNPs is challenging. The solution is to utilize the nonlinear magnetization on MNPs, and we can selectively detect only the MNPs in SLNs because other medical instruments do not possess nonlinear magnetization. This nonlinearity produces the harmonics signal with respect to applied AC magnetic fields for the magnetization of MNPs^37–40^. Under a DC magnetic field, the second harmonics signal could show the largest signal among the harmonics^40^.

In this study, we developed a novel magnetic probe, called an *ACDC-probe*, that utilizes alternating (AC) and direct (DC) magnetic fields to satisfy all surgical requirements and to solve the drawbacks of conventional methods. For the precise detection of only magnetic nanoparticles without the influences of other factors, such as those caused by other medical instruments, second harmonics signals of the nonlinear magnetization of the magnetic nanoparticles are detected under AC and DC magnetization. To generate the larger second harmonics signal strength, the ratio of AC and DC magnetic fields was optimized. Proximity detection of MNPs was experimentally evaluated compared to the *DC-probe*. We revealed that the influence of combined use of medical instruments significantly decreased. We also demonstrated the feasibility of laparoscopic SLN detection using the *ACDC-probe* in an animal experiment on a wild swine.

## Results and Discussion

### Magnetic technique for the laparoscopic detection of SLNs using a magnetic probe

**Figure 1:**
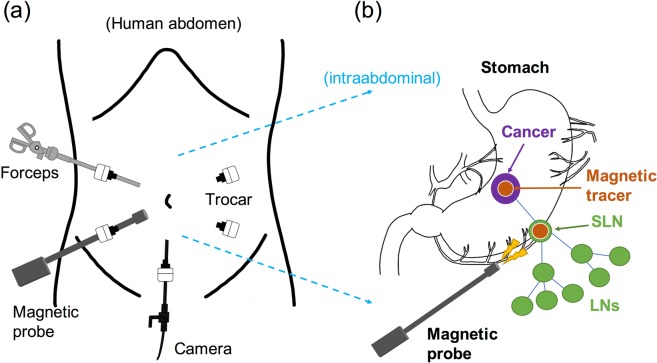
Magnetic technique for sentinel lymph node navigation in gastric cancer patients. (**a**) Medical instruments, such as forceps, camera, and a magnetic probe, are inserted using trocars. (**b**) Schematic of intraabdominal and laparoscopic identification of sentinel lymph nodes using a magnetic probe and magnetic tracers for gastric cancer patients. Sentinel lymph nodes containing magnetic tracers are detected by a magnetic probe laparoscopically.

To reduce the patient’s burden, trocars (typically five) are used for laparoscopic operation to minimize incision sizes/areas. Using trocars, medical instruments, such as forceps, a camera, and a dedicated probe to detect SLNs, are inserted as shown in Fig. 1(a). Figure 1(b) illustrates the newly developed magnetic technique of laparoscopic detection of gastric SLNs. We applied MNPs (magnetic tracer, Resovist®^41^) and a laparoscopic magnetic probe as an innovative technique.

**Figure 2:**
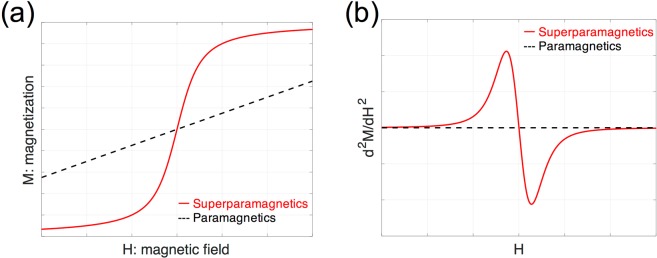
(**a**) Magnetization *M* of MNPs (red solid line) and typical paramagnetic materials (black dashed line) as a function of an applied external magnetic field *H*. (**b**) Nonlinearity of the *M-H* curve derived by second derivatives of *M-H* curve: MNPs (red solid line) and paramagnetism (black dashed line).

Figure 2 shows the principles behind the detection of magnetic nanoparticles using the *ACDC-probe*. MNPs have a superparamagnetic property, which is described by the Langevin function (Eq. (1)), as shown in Fig. 2(a). The magnetization *M* [A/m] of MNPs depends nonlinearly on an applied external magnetic field *H* [A/m]. In contrast, the magnetization of paramagnetic materials is linearly proportional to an external magnetic field. Note that this explanation omits the AC response of magnetic materials; detailed magnetic response with respect to AC magnetic fields must be considered magnetic relaxations, such as Néel and Brownian relaxations^39,42,43^. Figure 2(b) shows that the strength of the nonlinear response is represented by *d*^2^*M/dH*^2^. B. Gleich and J. Weizenecker reported that the nonlinearity produces harmonics signals (i.e., f_2_, f_3_, … f_n_) generated by magnetic nanoparticles with respect to the frequency f_0_ of the applied external magnetic field^37^. Under certain DC magnetic fields and AC fields the amplitude of the second harmonic signals could be the highest^40^. Therefore, we utilized the detection of the second harmonic signal. It should be noted that the paramagnetic case represents zero nonlinearity, as shown in Fig. 2(b), indicating that the harmonic signals would not be generated in the theoretical prediction.

1 $$M\propto \,\coth (H)-1/H.$$

### Developed *ACDC-probe* prototype utilizing nonlinear magnetic response of magnetic nanoparticles for laparoscopic detection

**Figure 3:**
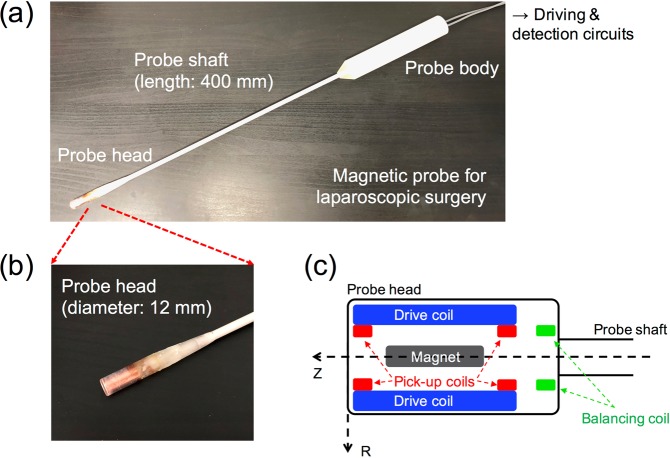
(**a**) *ACDC-probe* prototype for laparoscopic detection of sentinel lymph node containing magnetic nanoparticles. Length of probe shaft is 400 mm and electric cables are connected to driving and detection circuits (see Fig. 4). (**b**) Diameter of the probe head is 12 mm to be inserted through the trocar for laparoscopic operation. (**c**) Schematic of the probe head; a drive coil and permanent magnet generates AC and DC magnetic fields, respectively, for magnetization of magnetic nanoparticles. Newly generated magnetic fields of MNPs are measured by a detection coil (two pick-up coils and a balancing coil). Longitudinal direction of the probe is *Z*-axis and lateral direction is *R*-axis.

We developed a laparoscopic magnetic probe, *ACDC-probe*, utilizing AC and DC magnetic fields and evaluated its properties, such as the magnetic sensitivity, optimization of the ratio of AC to DC magnetic fields, detection length, and the detectable amount of iron for MNPs, as well as a comparison between the *ACDC-probe* and the previously developed *DC-probe*^26^. Figure 3 shows the detailed structure of the *ACDC-probe*. To enable insertion of the probe in the trocar for laparoscopic navigation, the outer diameter of the probe head and length of the probe shaft are 12 mm and 400 mm, respectively, as shown in Fig. 3(a). Figure 3(b,c) show an enlarged picture and schematics of the probe head, respectively, and the probe head consists of a coil system and a permanent magnet. Detailed characteristics of the drive solenoid coil and pick-up coil (balancing coil) are as follows (see Table 1): outer diameter is 10.5 mm and 6.7 mm, inner diameter is 7 mm and 3.5 mm, length is 23 mm and 3 mm, number of turns is 3000 and 1000, and wire diameter is 0.1 mm and 0.05 mm. The inductance is 23.5 mH and 3.6 mH and resistance is 192 Ω and 136 Ω. The outer diameter and length of the column-shaped permanent magnet (Neodymium: NF40) are 2 mm and 20 mm. The drive coil generates AC magnetic fields. The pick-up coils with opposite windings are connected in series as a gradiometer configuration, and the coils are located on both sides of the drive coil. The balancing coil is used for compensation of small errors in the gradiometer configuration, and the detection coil (10.8 mH and 408 Ω) is composed of the gradiometer and balancing coil. The permanent magnet (for generating DC magnetic fields) is located at the center of the drive coil. The directions along and perpendicular to the probe shaft are the *Z*-axis (longitudinal direction) and the *R*-axis (lateral direction), respectively.

**Table 1:** Details of coils and a permanent magnet consisting the *ACDC-probe*.

	Drive coil	Pick-up coil/balancing coil	Permanent magnet (NF40)
Outer diameter [mm]	10.5	6.7	2
Inner diameter [mm]	7	3.5	—
Length [mm]	23	3	20
Turns	3000	1000	—
Wire diameter [mm]	0.1	0.05	—
Inductance [mH]	23.5	3.6	—
Resistance [Ω]	192	136	—

### Driving and measurement system for AC magnetic field under the DC magnetic fields

**Figure 4:**
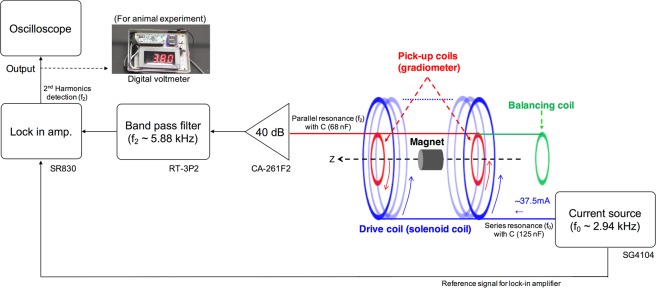
Schematic of driving and detection systems. A permanent magnet generates DC magnetic fields. Drive coil generates AC magnetic fields (f_0_ ~2.94 kHz) through a capacitance (~125 nF) for series resonance of f_0_; sine waves of 37.5 mA are produced by a function generator. Second harmonics signals (f_2_ ~5.88 kHz) detected through pick-up coils and a balancing coil is measured by lock-in amplifier via parallel resonance with a capacitance (~68 nF), a preamplifier (40 dB), and band pass filter (5.5–6.3 kHz). The digital voltmeter used for animal experiments detected the magnetic signal intensity and produced sounds based on the detected value (higher frequency sound for larger signal intensity).

Figure 4 shows the details of the driving and measurement system of the *ACDC-probe*. The driving coil produces AC magnetic fields with the frequency of f_0_ ~ 2.94 kHz. To generate the larger AC electric current (*I* ~37.5 mA), a current source, capacitance (~125 nF) for resonance, and drive coil are connected in series. DC magnetic fields are generated by a permanent magnet. MNPs are magnetized by AC magnetic fields under DC magnetic fields, and the detection coil (the gradiometer including a balancing coil) detects the magnetic fields generated by the magnetized MNPs. Two pick-up coils with opposite windings eliminate the detection of the excitation magnetic fields and the role of the balancing coil is to compensate the production error of the two pick-up coils. The inductive voltage generated by the time variations of the magnetic fields *B*_*Z*_ of the MNPs are measured by a lock-in detection system consisting of a pre-amplifier, bandpass filter, and lock-in amplifier. To make precise detections without the influence of other surgical instruments (paramagnetic material, e.g., a stainless-steel metal), second harmonics signal (f_2_ ~ 5.88 kHz) are measured via a parallel resonance (~68 nF). The voltmeter displays the magnetic signal intensity on its screen and produces a sound (a larger signal produces a higher frequency sound); thus, surgeons can judge whether lymph nodes are detected according to the value displayed and sound produced.

### Optimization of the ratio of AC and DC magnetic fields

**Figure 5:**
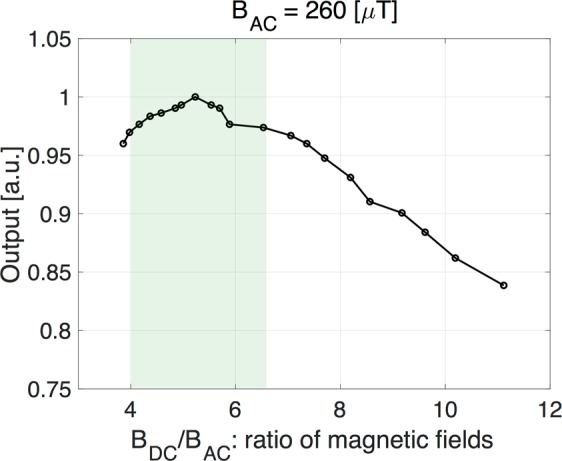
Normalized output signals versus the ratio of DC and AC magnetic fields *B*_DC_/*B*_AC_ with respect to the MNPs containing 140 μg of iron. Larger output signals were observed at *B*_DC_/*B*_AC_ = 4–6 as shown a green shaded area. The maximum value was observed at the ratio *B*_DC_/*B*_AC_ ~5. MNPs location is 3 mm from the probe head.

**Figure 6:**
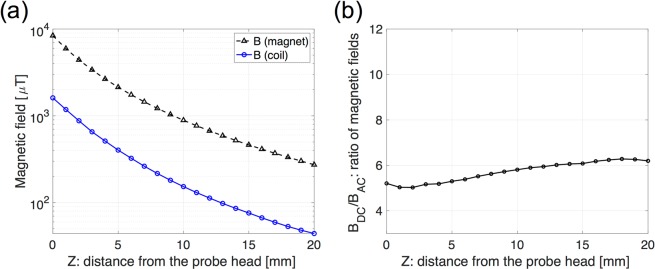
(**a**) Measured AC (blue solid line) and DC (black dashed line) magnetic fields as a function of distance from the probe head on *Z*-axis. (**b**) Ratio of *B*_DC_/*B*_AC_ as a function of the distance from the probe head on *Z*-axis. At *Z* = 0–20 mm, the ratio is approximately 5–6.

The intensity of the harmonic signals strongly depends on the magnetic field strength *B*_*Z*_, frequency, and the ratio of DC to AC magnetic fields^40^. The dependency on the ratio *B*_DC_/*B*_AC_ of second harmonics signal for 260 μT of AC fields is shown in Fig. 5. Under the relatively small static magnetic field, 1–2 mT, the measured signals of f_2_ from the magnetized MNPs (Resovist®) of 5 μL (140 μg iron) reached the maximum value at the ratio of *B*_DC_/*B*_AC_ ~5, which implies the presence of a large nonlinearity at this range. The detected signals decrease when increasing the ratio of *B*_DC_/*B*_AC_ ~ 5. To detect the larger signal (>95%), the ratio of *B*_DC_/*B*_AC_ should be kept within the range from 4 to 6. Figure 6(a) shows the distribution of measured AC and DC magnetic fields of the *Z*-component generated by the drive coil and the magnet, respectively, on the *Z*-axis. The range of *B*_DC_/*B*_AC_ is from 5 to 6 as shown in Fig. 6(b).

### Detection distance and detectable iron amount for magnetic nanoparticles

**Figure 7:**
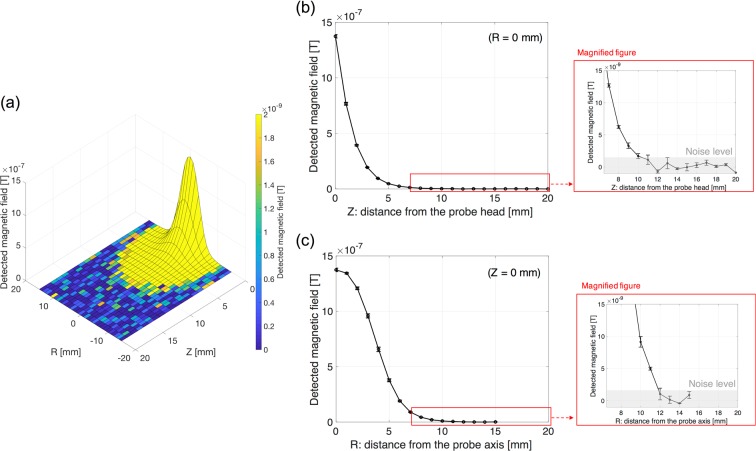
(**a**) Two-dimensional, (**b**) longitudinal (*R* = 0 mm), and (**c**) lateral (*Z* = 0 mm) detection length with respect to MNPs of 5 μL (140 μg iron). Enlarged view on (**b**,**c**) represents a longitudinal and lateral detection length of approximately 10 and 11 mm, respectively. Error bar represents an average of three measurements. Noise level is approximately 1 nT.

**Figure 8:**
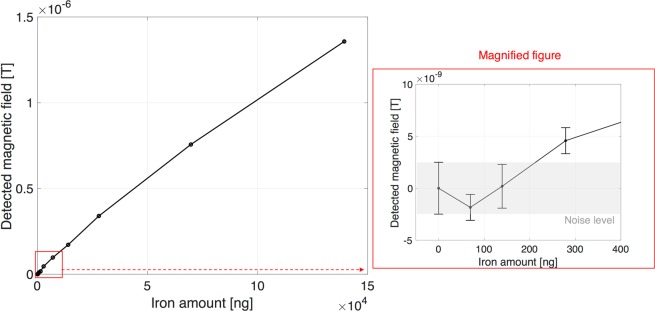
Detection limit of the amount of iron located 1 mm apart from the probe head is approximately 280 ng. Error bar represents an average of three measurements (standard deviation is approximately 1 nT).

Figure 7(a) shows the two-dimensional sensitivity mapping generated by the magnetized MNPs (as Fe of 140 μg) as a function of distance from the probe head. The detected magnetic signal decreases with increasing distance, and the minimum strength of detectable magnetic fields is approximately 1 nT (noise level). The longitudinal and lateral detection length is approximately 10 and 11 mm as shown in the magnified view of Fig. 7(b,c), respectively. Figure 8 shows the detectable amount of iron in MNPs 1 mm apart from the probe head and the *ACDC-probe* can detect as little as 280 ng of iron.

Currently, although there is no clear evidence about the depth of SLNs and iron amount in SLNs for gastric cancer patients, we previously revealed the iron amount in SLNs of breast cancer patients is 140 ± 80 μg and detected the MNPs in the SLNs using *DC-probe*^26^. In this previous research, the transcutaneous detection from the patient’s skin showed difficulties in some cases because the distance between SLNs and the skin surface might be long. However, in terms of laparoscopic detection, the probe could directly approach the connective fat tissue of the gastric SLNs at a shorter distance. We believe that the detection length and detectable iron amount is sufficient for laparoscopic detection with regards to gastric cancer patients.

### Proximity detection for laparoscopic operation

So far, for conventional laparoscopic SLNB using the RI method in the limited intraabdominal space, it is surgically difficult to intraoperatively sample SLNs. The large radioactive signal from the primary tumor site occasionally might prevent SLN detection when the locations of SLNs is in close proximity to the primary tumor (injection site); surgeons calls this phenomenon the “shine-through effect”^14,16^. As the shine-through effect restricts SLN detection, the RI method is generally available for only the confirmation of complete SLN harvest^15^. Thus, it is necessary to detect SLNs without the influence of the shine-through effect, which is proximity-based detection.

**Figure 9:**
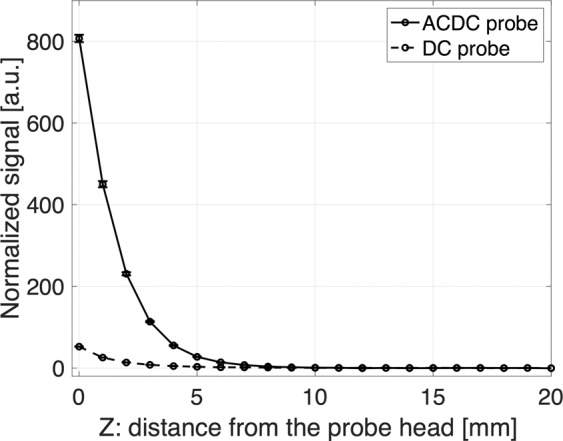
Detection performance of *ACDC-probe* (solid line) compared with *DC-probe*^26^ (dashed line) as a function of distance from the probe head on *Z*-axis. Detected signals from the MNPs (140 μg of iron) are normalized by the noise level of each probe (~1 nT for the *ACDC-probe* and ~1 μT for the *DC-probe*). Detailed of the *DC-probe* are described in ref. ^26^. Error bar represents an average of three measurements.

Figure 9 shows the comparison between *ACDC-probe* and *DC-probe*^18^ regarding the signal normalized by the noise level of each probe for the proximity-based detection; the noise level is ~1 nT for the *ACDC-probe* and ~1 μT for the *DC-probe*). The signals detected by the *ACDC-probe* significantly increase with decreasing distance from the probe head compared with *DC-probe*, and the largest values of *ACDC-probe* and *DC-probe* are approximately 800 and 50 times larger than the detection limit, respectively. These results suggest that *ACDC-probe* enables us to provide proximity detection compared with *DC-probe*. Regarding *DC-probe*, the MNPs are fully magnetized with >50 mT of DC magnetic fields in *Z* = 0–10 mm^26^ so the magnetization of the MNPs does not produce larger differences in this distance range. On the other hand, the MNPs closer to the *ACDC-probe* produce larger magnetization because the AC and DC magnetic fields, *B*_AC_ ~1.6 mT and *B*_DC_ ~8.4 mT at *Z* = 0 mm, as shown in Fig. 6(a), are not large enough to saturate the magnetization, providing the proximity-based detection capability of the *ACDC-probe*.

### Influences of combined used medical instruments and biomedical tissues

**Figure 10:**
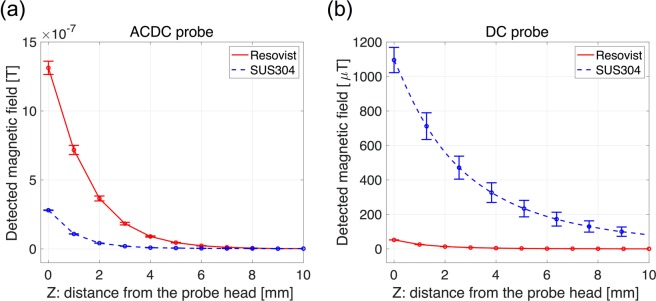
Comparison between (**a**) *ACDC-probe* and (**b**) *DC-probe* with respect to the detection of MNPs (red solid line: 140 μg of iron) and SUS304 (blue dashed line: a typical stainless-steel metal for medical instruments). Error bar represents an average of three measurements.

For laparoscopic operations, we also have to consider the influences of the combined use of other medical instruments for magnetic detection because most of the typical instruments are made of magnetic materials (metals). For instance, stainless-steel (e.g., SUS304) is a commonly used material because of its biocompatibilities. The metal has paramagnetic properties (a tiny amount of ferromagnetism in some cases). In addition, we evaluated the influence of the biomedical tissues, which exhibit diamagnetism, using saline. In general, the magnetic characteristics of the material is linearly proportional to the external magnetic fields. As mentioned in introduction, the MNPs have the superparamagnetic property with nonlinear magnetization with respect to the external magnetic field. The concept of *ACDC-probe* extracts only the nonlinear characteristics of the MNPs and eliminates undesirable effects contributed by paramagnetism as well as diamagnetism. Figure 10(a,b) show the signal strength with respect to the MNPs (140 μg Fe) and SUS304 pipe (diameter: 5 mm and length 400 mm) detected by the *ACDC-probe* and *DC-probe*. Significant differences in the detection characteristics of both probes could clearly be observed; the largest signal was measured on Resovist® in the *ACDC-probe* and on SUS304 in the *DC-*probe. The MNPs/SUS304 is located 1 mm apart from the probe head. For *ACDC-probe*, the detected signal of SUS304 is approximately 0.2 of the normalized signal detected by the MNPs. On the other hand, a much larger signal (~21) is seen in the detection using *DC-probe*. We note that the detected signal from the paramagnetic material should ideally be zero. However, the detected signal was not zero because the metal under AC magnetic fields generates new AC magnetic fields due to the eddy current leading to the variation of the magnetic fields or small amount of ferromagnetism with nonlinear magnetization. The signal intensities detected by both probes in the experiment using saline is comparable to the noise level (see Supplementary Information). In terms of the longer distance detection of SUS304 (10 mm apart from the probe head), the signal intensity of *ACDC-probe* is as small as its noise level (~1 nT). In contrast, the signal intensity of *DC-probe* is still much larger, showing >80 μT, than its noise level of ~1 μT. The detectable distance of *ACDC-probe* with respect to SUS304 is much shorter than that of the *DC-probe*; the distances of *ACDC-* and *DC-probe* are approximately 10 and 42 mm, respectively. These results indicate that the *ACDC-probe* can minimize the influence of other surgical instruments and biomedical tissues.

### Laparoscopic detection with magnetic tracer and magnetic probe in animal experiment

We performed an animal experiment on a swine at Keio University in Japan, to verify the feasibility of the developed laparoscopic magnetic probe. The magnetic tracers (1.0 mL of diluted magnetic tracers: Resovist®^41^ 0.5 mL and saline 0.5 mL) were endoscopically injected in the submucosal layer of a simulated cancer location (i.e., the greater curvature of the lower third of the stomach) by using an endoscopic puncture needle.

**Figure 11:**
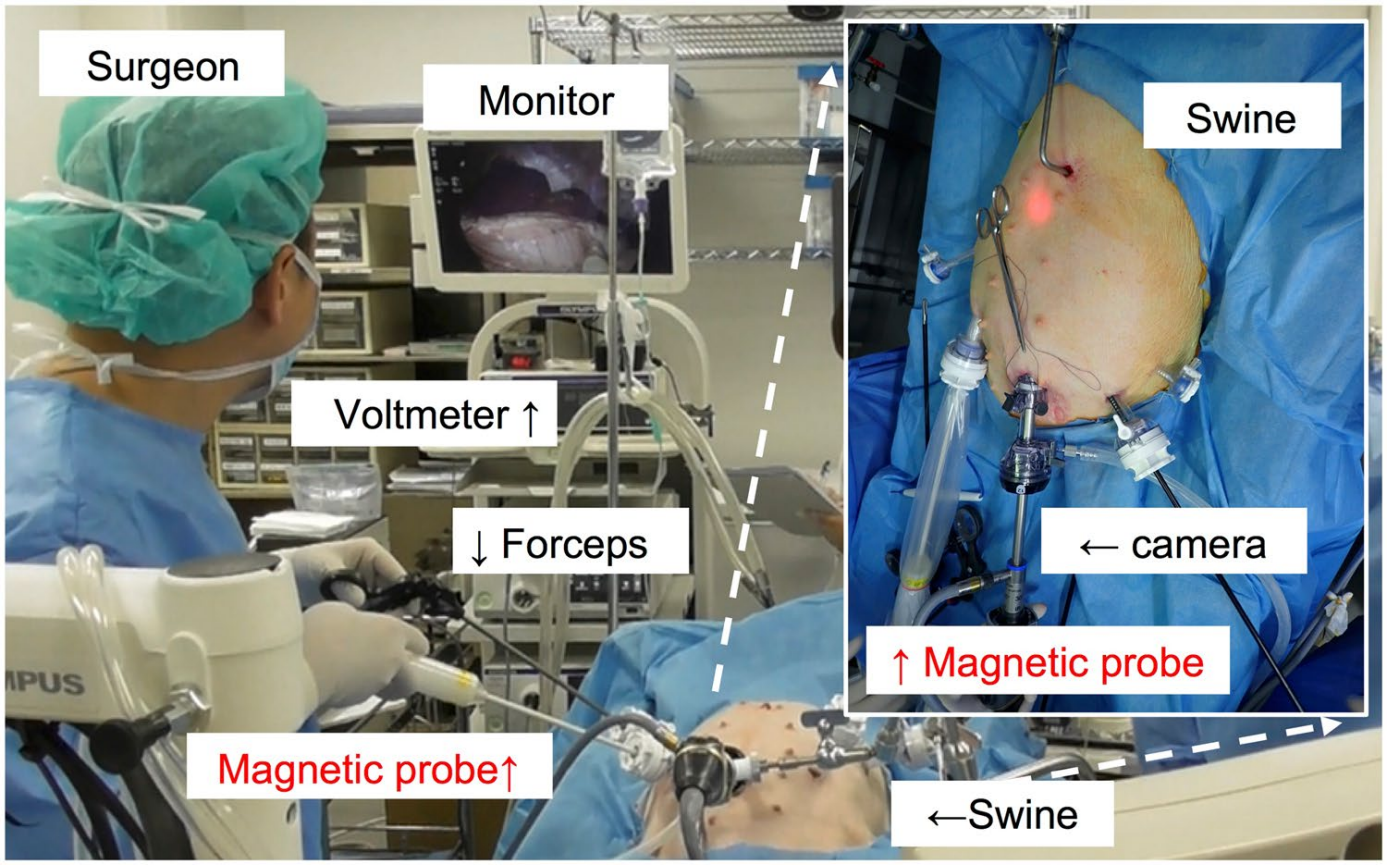
Animal experiment of laparoscopic detection involving developed *ACDC-probe* prototype in a swine (a wild-type swine, weight: 26.4 kg). Surgeon is finding LNs containing the MNPs by means of the laparoscopic instruments via trocars under the monitoring of laparoscopic camera in accordance with the displayed value and sound on voltmeter.

**Figure 12:**
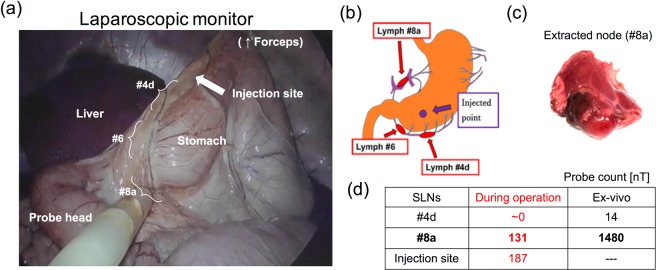
(**a**) Laparoscopic detection of SLN (#8a) using *ACDC-probe* prototype in the laparoscopic surgery. SLN (#8a) was detected by the probe, and the stomach was lifted up by forceps to find the SLNs. (**b**) Schematic of SLNs location in a stomach^34^. (**c**) Excised lymph node with brownish-color of MNPs. (**d**) Magnetic strength of SLNs in laparoscopic detection (during operation) and *ex-vivo* (after extraction) detection.

After injection of MNP tracers, it takes typically 15 minutes to accumulate into the lymph nodes. The surgeons subsequently inserted the *ACDC-probe* prototype via a trocar and tried to detect the lymph nodes containing the MNPs via laparoscopic observations as shown in Fig. 11. Figure 12(a) shows that the surgeons succeeded in finding the gastric SLN (#8a^44^ as shown in Fig. 12(b)) by the *ACDC-probe* for 27–37 minutes after injection of MNPs; the strength of the magnetic signal was approximately 131 nT in the laparoscopic detection. In terms of the injection site, the magnetic strength showed 187 nT, which is larger than that of the SLN (#8a). However, it was clearly separate from the detection of the SLN (#8a) and injection site, indicating there is no shine-through effect. Regarding the LN (#4d), we did not detect in the laparoscopic investigation, which indicates that this LN is not an SLN. After the laparoscopic detection, gastric SLNs were excised. Extracted SLN (#8) showed the brownish-color at the location accumulating with MNPs as shown in Fig. 12(c). In the *ex-vivo* evaluations (Fig. 12(d)), the SLNs (#8a) show about 10 times larger magnetic strength, reading 1480 nT, compared with the intraoperative detection. This result indicates that smaller magnetic signals were observed in the laparoscopic operation due to the longer distance originating from the thickness of connective fat tissues between the probe head and the SLNs. Consequently, these results suggest that we verified the feasibility of the laparoscopic SLN detection using the magnetic probe in a swine.

## Conclusions

We developed a magnetic probe for identifying SLNs containing magnetic nanoparticles in laparoscopic operation of gastric cancer patients. To detect only the second harmonics generated by the nonlinear magnetization of the magnetic nanoparticles, the *ACDC-probe* prototype was comprised of the drive coil for AC magnetization and permanent magnet for DC magnetization. The ratio of *B*_DC_ to *B*_AC_ was optimized (*B*_DC_/*B*_AC_ ~ 5) for the generation of a larger second harmonics signal. The longitudinal detection length is approximately 10 mm for 140 μg of iron, and the detectable amount of iron is approximately 280 ng at a distance of 1 mm. Furthermore, the proximate-based detection was demonstrated and the influence of stainless-steel, which is typically used for medical instruments, was significantly reduced from the measured signals compared with the *DC-probe*. These results indicate that the newly developed *ACDC-probe* is effective for identifying the SLNs in laparoscopic surgery. We conducted an animal study on the use of the laparoscopic magnetic probe and magnetic tracers in a swine to verify the performance of magnetic detection. The SLN containing the magnetic nanoparticles was clearly detected without the influence of the shine-through effect from the injection site in the laparoscopic searching. To conclude, the newly developed magnetic probe was found to be potentially beneficial for the identification of the SLNs.

Further studies, such as the optimization of the frequency for harmonics response, will be performed for the highly sensitive magnetic detection of much smaller accumulations in SLNs. Higher frequency generates larger voltage based-on Faraday’s law. However, at higher frequency, the magnetization of the magnetic nanoparticles strongly depends on Brownian and Néel relaxation^38,39^, and the magnetization strength could decrease, which indicates that there could be an optimum frequency for detecting the magnetic nanoparticles. Furthermore, from a clinical viewpoint, the iron quantity in extracted nodes will be revealed by iron quantification device^45^ for future human operations because the minimization of the injection amount of magnetic nanoparticles is required for clinical usages. In the near future, we plan to introduce the magnetic probe to hospitals for a clinical trial and will demonstrate the magnetic SLN navigation in gastric cancer patients.

## Materials and Methods

### Resovist® (magnetic tracers containing superparamagnetic iron oxide nanoparticles)

Resovist®^41^ was originally developed as a contrast agent for contrast-enhanced magnetic resonance imaging (MRI) and is clinically approved superparamagnetic iron oxide nanoparticles coated by carboxydextran with a diameter of ~60 nm for identifying liver cancers. Although this magnetic tracer does not have the function actively up-taken into carcinogenic cells of LNs, the macrophages in the LNs takes the magnetic tracer in the lymphatic systems, resulting in the tracer accumulation into the LNs. The iron concentration is approximately 28 mg/mL. In this study, we used Resovist® as a magnetic tracer. However, Resovist® is clinically approved only in limited counties (e.g., Japan). In other countries, such as Europe and USA, Sienna+®^6^ (which has the same concentration of iron as Resovist®) is the clinically approved tracer with which patients can receive the proposed SLNB.

### Laparoscopic magnetic probe, *ACDC-probe*

A neodymium magnet (NF40, with typical coercive force of approximately 950–1000 kA/m) manufactured by Magfine Co., Ltd., Japan was used for the generation of the DC magnetic fields. A driving solenoid coil was manufactured by Nihon Universal Electric Co., Ltd., Japan for the generation of the AC magnetic fields. Pickup coils and a balancing coil also were manufactured by Nihon Universal Electric Co., Ltd., Japan for the detection of the magnetic fields newly generated by the SPIONs magnetized by the AC fields under the DC fields. The applied AC voltage and current (SG-4104, IWATSU ELECTRIC CO. LTD., Japan) was approximately 10 V and 37.5 mA, respectively. The capacitance (~125 nF) is connected with the drive coil in series to increase the electric current. The outer diameter of the probe head and the length of the probe shaft was 12 mm and 400 mm, respectively. The probe housing was made of acrylonitrile-butadiene-styrene (ABS) resin. The surface temperature of the probe head measured by thermography (InfReC Thermo GEAR G100, Nippon Avionics Co., Ltd., Japan) is approximately less than 41 °C (typically 35–40 °C at room temperature: ~20–25 °C), indicating that the probe can be used for humans. To increase the rigidity, there is a brass pipe inside the probe shaft. Highly sensitive electric circuits, consisting of a resonance capacitance (~68 nF), preamplifier (CA-261F2; NF Corporation, Japan), bandpass filter (RT-3P2; NF Corporation, Japan), and Lock-in-amplifier (SR830; Stanford Research Systems, USA), were used to measure the small magnetic signals. The detected signal was displayed on the LED display panel in real time during the laparoscopic identification and *ex-vivo* experiments.

### Measurement of DC and AC magnetic fields

The distribution of DC and AC magnetic fields on the probe axis (*Z*-axis) was measured by the Hall sensor (A1389 manufactured by Allegro MicroSystems, LLC, USA) and by using the magnetic field mapping system^20^.

### Measurements of AC response of MNPs under DC magnetic fields

Magnetic signals generated by 5 μL of Resovist (as 140 μg of iron) were measured under the constant AC fields (260 μT) and varied DC magnetic fields. The constant AC magnetic fields were generated by the driving coil (37.5 mA). Varied DC magnetic fields (1–2 mT) were applied to the SPIONs by changing the distance between a permanent magnet and the probe head. We conducted on the experiments from much larger DC fields (~1 mT) compared with geomagnetic fields (40–50 μT depending on locations) to minimize the influence of geomagnetic fields.

### Experimental evaluations of the developed *ACDC-probe*

In terms of the detection distance, magnetic signals generated by 5 μL of Resovist® (140 μg of iron) were measured on the probe axis with different distances between probe head and Resovist. For the detection limit of the iron amount, magnetic phantoms, except for neat Resovist® (140 μg of iron), were fabricated by the dilution of Resovist; solvent is pure water to prevent particle aggregations. The volume of all phantoms was 5 μL and the phantom was located 1 mm from the probe head. To evaluate a metal phantom, SUS304 pipe (stainless-steel metal: diameter is 5 mm, thickness is 0.5 mm, and length 400 mm) were used for simulated medical instruments. SUS304 pipe was located with vertically oriented with respect to the probe axis (*Z*-axis). To simulate biomedical tissues with diamagnetism, measurements were conducted using 1 L of saline (detailed descriptions are in Supplementary Information).

### Animal experiments for demonstration of the feasibility of the laparoscopic magnetic probe

To verify the feasibility of the developed magnetic probe system, an animal experiment was conducted on a wild-type swine (weight: 26.4 kg) in accordance with the protocols for the care of animals and scientific purposes. The protocols were approved by the ethical community of Keio University (approval number: 08073). 0.5 mL of Resovist® with 0.5 mL of saline (1 mL of diluted magnetic tracer: iron concentration is ~14 mg/mL) was endoscopically injected in the submucosal layer of a simulated cancer location (e.g., the greater curvature of the lower third of the stomach) using an endoscopic puncture needle. We used diluted Resovist® because the dilution could promote the tracer accumulation into lymph nodes^13^. We confirmed that there were no additional LNs containing MNPs in the entire area of the stomach by using the developed magnetic probe after the resection of the stomach (*ex-vivo* detection).

## Supplementary information

Supplementary information.

## References

[1] Krag DN, Weaver DL, Alex JC, Fairbank JT (1993). Surgical resection and radiolocalization of the sentinel lymph node in breast cancer using a gamma probe. Surg Oncol..

[2] Giuliano AE, Kirgan DM, Guenther JM, Morton DL (1994). Lymphatic mapping and sentinel lymphadenectomy for breast cancer. Ann. Surg..

[3] Kitagawa Y, Kitajima M (2004). Sentinel node mapping for gastric cancer: is the jury still out?. Gastric Cancer.

[4] Ahmed M, Purushotham AD, Douek M (2014). Novel techniques for sentinel lymph node biopsy in breast cancer: a systematic review. Lancet Oncol..

[5] Shiozawa M (2013). Sentinel lymph node biopsy in patients with breast cancer using superparamagnetic iron oxide and a magnetometer. Breast Cancer..

[6] Douek M (2014). Sentinel Node Biopsy Using a Magnetic Tracer versus Standard Technique: The SentiMAG Multicentre Trial. Ann. Surg. Oncol..

[7] Thill M (2014). The Central-European SentiMag study: Sentinel lymph node biopsy with superparamagnetic iron oxide (SPIO) vs. radioisotope. The Breast..

[8] Rubio IT (2015). The superparamagnetic iron oxide is equivalent to the Tc99 radiotracer method for identifying the sentinel lymph node in breast cancer. Eur. J. Surg. Oncol..

[9] Pienero-Madrona A (2015). Superparamagnetic iron oxide as a tracer for sentinel node biopsy in breast cancer: A comparative non-inferiority study. Eur. J. Surg. Oncol..

[10] Ghilli M., Carretta E., Di Filippo F., Battaglia C., Fustaino L., Galanou I., Di Filippo S., Rucci P., Fantini M.P., Roncella M. (2015). The superparamagnetic iron oxide tracer: a valid alternative in sentinel node biopsy for breast cancer treatment. European Journal of Cancer Care.

[11] Houpeau J (2016). Sentinel Lymph Node Identification Using Superparamagnetic Iron Oxide Particles Versus Radioisotope: The French Sentimag Feasibility Trial. J. Surg. Oncol..

[12] Zada A (2016). Meta-analysis of sentinel lymph node biopsy in breast cancer using the magnetic technique. Br. J. Surg..

[13] Kuwahata A (2018). Combined use of fluorescence with a magnetic tracer and dilution effect upon sentinel node localization in a murine model. International Journal of Nanomedicine.

[14] Kitagawa Y (2013). Sentinel Node Mapping for Gastric Cancer: A prospective Multicenter Trial in Japan. Journal of Clinical Oncology.

[15] Kitagawa Y (2005). Minimally invasive surgery for gastric cancer – toward a confluence of two major streams: a review. Gastric Cancer.

[16] Takeuchi H (2011). Laparoscopy-assisted Proximal Gastrectomy with Sentinel Node Mapping for Early Gastric Cancer. World J. Surg..

[17] Takeuchi H, Kitagawa Y (2013). New Sentinel Node Mapping Technologies for Early Gastric Cancer. Ann. Surg. Oncol..

[18] Takeuchi H, Kitagawa Y (2013). Sentinel Node Navigation Surgery in Patients with Early Gastric Cancer. Dig. Surg..

[19] Arigami T (2017). Clinical application and outcomes of sentinel node navigation surgery in patients with early gastric cancer. Oncotarget.

[20] Gotoda T (2000). Incidence of lymph node metastasis from early gastric cancer: estimation with a large number of cases at two large centers. Gastric Cancer.

[21] Lee YJ (2015). Prospective multicenter feasibility study of laparoscopic sentinel basin dissection for organ preserving surgery in gastric cancer. Medicine.

[22] Shida A (2018). Sentinel node navigation surgery for early gastric cancer: Analysis of factors which affect direction of lymphatic drainage. World J. Surg..

[23] Shoji Y (2019). Prospective feasibility study for single-tracer sentinel node mapping by ICG (indocyanine green) fluorescence and OSNA (one-step nucleic acid amplification) assay in laparoscopic gastric cancer surgery. Gastric Cancer.

[24] Feng JW (2019). Comparison of laparoscopic and open approach in treating gallbladder cancer. J. Surg. Res..

[25] Bray F (2018). Global cancer statistics 2018: GLOBOCAN estimates of incidence and mortality worldwide for 36 cancers in 185 Countries. CA: Cancer J. Clin..

[26] Sekino M (2018). Handheld magnetic probe with permanent magnet and Hall sensor for identifying sentinel lymph nodes in breast cancer patients. Scientific Reports.

[27] Ookubo T (2013). Characteristics of magnetic probes for identifying sentinel lymph nodes. Conf. Proc. IEEE Eng. Med. Biol. Soc..

[28] Kuwahata A (2017). Three-dimensional sensitivity mapping of a handheld magnetic probe for sentinel lymph node biopsy. AIP Adv..

[29] Kaneko M (2017). A magnetic probe equipped with small-tip permanent magnet for sentinel lymph node biopsy. AIP Adv..

[30] Pouw JJ, Bastiaan DMC, Klaase JM, ten Haken B (2016). Phantom study quantifying the depth performance of a handheld magnetometer for sentinel lymph node biopsy. Physica Medica.

[31] Harvey JR (2018). Safety and feasibility of breast lesion localization using magnetic seeds (Magseed): a multi-centre, open-label cohort study. Breast Cancer Research and Treatment.

[32] Pohlodek K, Foltín M, Mečiarová I, Ondriaš F (2018). Simultaneous use of magnetic method in localization of impalpable breast cancer and sentinel lymph nodes detection: initial experience. Nanomedicine (London).

[33] Waanders S (2016). A handheld SPIO-based sentinel lymph node mapping device using differential magnetometry. Phys. Med. Biol..

[34] Visscher M, Waanders S, Krooshoop HJG, ten Haken B (2014). Selective detection of magnetic nanoparticles in biomedical applications using differential magnetometry. Journal of Magnetism and Magnetic Materials.

[35] Karo H, Sasada I (2017). Superparamagnetic nanoparticle detection system by using a fundamental mode orthogonal fluxgate (FM-OFG) gradiometer. AIP Adv..

[36] Cousins A (2018). Novel handheld magnetometer probe based on magnetic tunnelling junction sensors for intraoperative sentinel lymph node identification. Scientific Reports.

[37] Gleich B, Weizenecker J (2005). Tomographic imaging using the nonlinear response of magnetic particles. Nature.

[38] Mamiya H, Jeyadevan B (2015). Nonequilibrium Magnetic Response of Anisotropic Superparamagnetic Nanoparticles and Possible Artifacts in Magnetic Particle Imaging. PLoS ONE.

[39] Rosensweig RE (2002). Heating magnetic fluid with alternating magnetic field. Journal of Magnetism and Magnetic Materials.

[40] Weaver JB, Rauwerdink AM, Sullivan CR, Baker I (2008). Frequency distribution of the nanoparticle magnetization in the presence of a static as well as a harmonic magnetic field. Med. Phys..

[41] Reimer P, Balzer T (2002). Ferucarbotran (Resovist): a new clinically approved RES-specific contrast agent for contrast-enhanced MRI of the liver: properties, clinical development, and applications. Eur. Radiol..

[42] Deissler RJ, Wu Y, Martens MA (2014). Dependence of Brownian and Néel relaxation times on magnetic field strength. Med. Phys..

[43] Yoshida T, Nakamura T, Higashi O, Enpuku K (2018). Magnetic fractionation and characterization of magnetic nanoparticles for magnetic particle imaging. Japanese Journal of Applied Physics.

[44] Japanese Gastric Cancer Association (2011). Japanese classification of gastric carcinoma: 3^rd^ English edition. Gastric Cancer.

[45] Kuwahata A (2018). Development of device for quantifying magnetic nanoparticle tracers accumulating in sentinel lymph nodes. AIP. Adv..

