# Supplementary material for: Development of Magnetic Probe for Sentinel Lymph Node Detection in Laparoscopic Navigation for Gastric Cancer Patients
Source: Sci Rep. 2020 Feb 4;10:1798. doi: 10.1038/s41598-020-58530-5 (PMC7000689; doi:10.1038/s41598-020-58530-5)
Supplement: Supplementary file 1 — Supplementary information. [file 41598_2020_58530_MOESM1_ESM.pdf]

Supplementary information

Development of Magnetic Probe for Sentinel Lymph Node Detection in Laparoscopic Navigation for Gastric Cancer Patients

Akihiro Kuwahata[1], Ryo Tanaka[1], Sachiko Matsuda[2], En Amada[2], Tomoyuki Irino[2], Shuhei Mayanagi[2], Shinichi Chikaki[1], Itsuro Saito[4], Norio Tanabe[1], Hirofumi Kawakubo[2], Hiroya Takeuchi[3], Yuko Kitagawa[2], Moriaki Kusakabe[5,6], and Masaki Sekino[1]

[1]Graduate School of Engineering, The University of Tokyo, Tokyo 113-0032, Japan
[2]Department of Surgery, Keio University School of Medicine, Tokyo, 160-8582, Japan
[3]Department of Surgery, Hamamatsu University School of Medicine, Hamamatsu, 431-3192, Japan
[4]iMed Japan Inc., Chiba, 275-0001, Japan
[5]Research Center for Food Safety, Graduate School of Agricultural and Life Sciences, The University of Tokyo, Tokyo 113-8657, Japan
[6]Matrix Cell Research Institute Inc., Ibaraki 300-1232, Japan
Correspondence and requests for materials should be addressed to A. K. (kuwahata@bee.t.u-tokyo.ac.jp) and M. S. (sekino@bee.t.u-tokyo.ac.jp).

Influences of biomedical tissues on detection

We evaluated the influence of biomedical tissues, which exhibit diamagnetism, using saline (water containing 0.9% of NaCl). In general, the magnetic characteristic of saline is linearly and inversely proportional to the external magnetic fields. The concept of the *ACDC-probe* extracts only the nonlinear characteristics of the MNPs and eliminates undesirable effects contributed by diamagnetism (as well as paramagnetism).

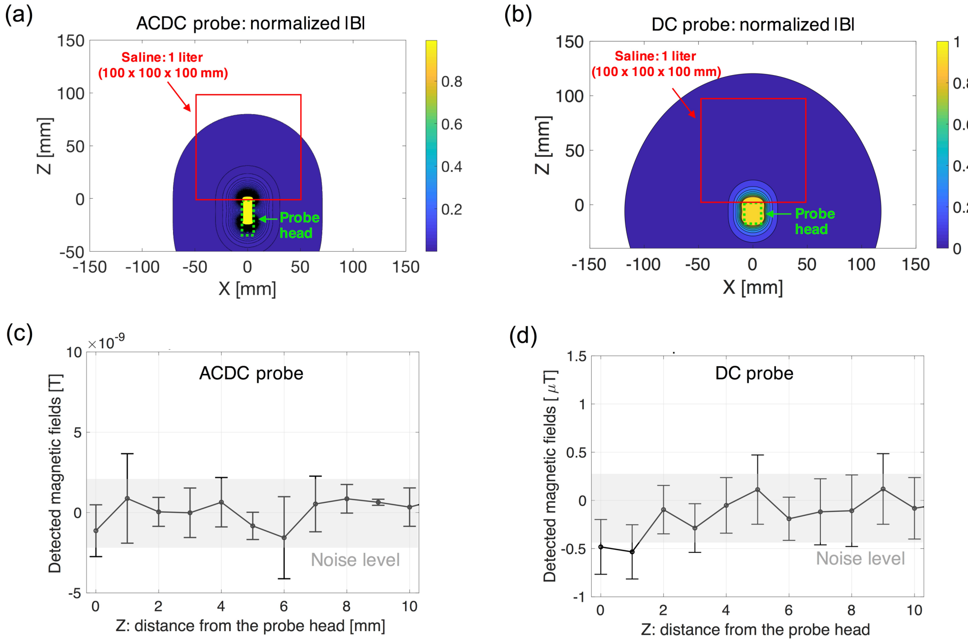

Supplementary Figure S1. Experiment using 1 L of saline. (a, b) Magnetic field strength |B| normalized by the strength at the location of the probe head (at *Z* = *X* = 0): (a) *ACDC-probe* and (b) *DC-probe*. Red solid-line area represents 1 L of saline, and green dotted-line area represents probe head. (c, d) Detected magnetic fields as a function of the distance of probe head: (c) *ACDC-probe* and (d) *DC-probe*. Error bar represents an average of three measurements.

To simulate a human body (biomedical tissue), we performed the experiment with 1 L (0.1 × 0.1 × 0.1 m^3) of saline. Although the typical volume required for the human body is ~80 L (~0.3 × 0.16 × 1.7 m^3), 1 L of saline is sufficient because it can be used to simulate 80 L of saline. As shown in Figs. S1(a) and (b), the strength of magnetic fields of *ACDC-* and *DC-probes* rapidly decreases with increasing the distance from the probe head The strength is mainly less than 1/1000 outside the volume of 1 L saline (outside the red solid-line area in Figs. S1(a) and (b)). This indicates the effect originated outside the area of saline is also less than 1/1000. Even if we use 80 L of saline, the effect can be negligibly small.

Figures S1(c) and (d) show detected magnetic fields as a function of the distance from the probe head. The detected magnetic fields using the *ACDC-/DC-probe* were still comparable to the noise level. Regarding the *ACDC-probe*, we considered two reasons behind this phenomenon: (1) our

system can detect only the second harmonics signal originated from the nonlinear magnetization of materials. However, the magnetization of saline is linear ideally. Thus, our system cannot detect the effect of saline. (2) The electrical conductivity of saline (~1.6 S/m) is much smaller than that of stainless steel (~1.4×10^6 S/m), indicating that the effect of eddy current is negligibly small when compared with stainless steel.
